# Enhancing cognitive functions in aged dogs and cats: a systematic review of enriched diets and nutraceuticals

**DOI:** 10.1007/s11357-025-01521-z

**Published:** 2025-01-18

**Authors:** Tiphaine Blanchard, Justine Eppe, Amélie Mugnier, Fabienne Delfour, Annabelle Meynadier

**Affiliations:** 1 GenPhySE, Université de Toulouse, INRAE, ENVT, 31326 Castanet Tolosan, France; 2 Lallemand SAS, 31700 Blagnac, France; 3 Clinical Department of Production Animals, Fundamental and Applied Research for Animals & Health Research Unit (FARAH), Faculty of Veterinary Medicine, University of Liège, Liege, Belgium; 4 ILCB- Aix Marseille Université, 13284 Marseille, France; 5 Service Alimentation – ENVT, 23 Chemin Des Capelles, 31300 Toulouse, France

**Keywords:** Aging, Canine, Cognition, Feline, Food additives, Nutrition therapy

## Abstract

With advancements in veterinary care and the growing recognition of pets as integral member of the family, the lifespans of dogs and cats have significantly increased, leading to a higher prevalence of age-related conditions, including cognitive dysfunction syndrome (CDS). CDS adversely impacts pets’ quality of life and presents emotional and practical challenges for owners. Given its similarities to Alzheimer’s disease in humans, CDS has gained attention as a target for nutrition-based interventions aimed at preserving cognitive function. This systematic review evaluates the efficacy of enriched diets and nutraceuticals in improving cognition in aging companion animals. A literature search was conducted using PubMed, CAB Abstracts, Web of Science, and Dimensions to identify clinical trials published in English that investigated the effects of enriched diets or nutraceuticals on cognitive functions in aged cats or dogs. Study quality was assessed using a modified CAMARADES checklist. A total of 30 studies (27 canine and 2 feline trials) published between 2002 and 2023 were reviewed. Studies on enriched diets generally demonstrated higher methodological quality compared to those on supplements. Omega-3 fatty acids showed cognitive benefits in aging pets, especially at higher doses, while antioxidants from plant extracts and products and vitamins E and C alone were less effective but remain essential for stabilizing omega-3 fatty acids. Other supplements, including S-adenosyl methionine, medium-chain triglycerides, homotaurine, and apoaequorin, also showed promise. However, future studies must standardize protocols, include robust control groups, and utilize both objective tasks and subjective questionnaires to strengthen conclusions.

**Supplementary Information:**

The online version contains supplementary material available at 10.1007/s11357-025-01521-z.

## Introduction

With improvements in veterinary care [1], and owners increasingly considering their pets as family members [2, 3], the lifespans of dogs and cats have increased [4]. This has led to a rise in age-related diseases [5], globally the same as for humans [1, 6], extending the concept of “health span” to pets [7]. Aging is a progressive and intrinsic decline in physiological functions, influenced by toxic environmental factors and limited genomic adaptability [8]. This decline compromises homeostasis and responsiveness to the environment [9–11], reducing pets’ autonomy and burdening owners [12, 13].

Among these declining functions, cognitive dysfunction is particularly impactful [14], leading to cognitive dysfunction syndrome (CDS) in dogs and cats [15]. Canine CDS prevalence increases with age and may begin as early as 6 years old [16], affecting 14–35% of dogs by age 8 [17]. Feline CDS is less studied [18]. However, one-third of cats aged 11–14 years show symptoms, with prevalence increasing with age [19]. Clinical signs of CDS in both species are grouped into six categories: *D*isorientation, altered social *I*nteractions, changes in *S*leep–wake cycles, loss of *H*ousetraining, altered *A*ctivity levels, and increased *A*nxiety; defining “DISHAA” approach [15].

CDS is linked to several changes in the aging brain [17, 18]. Briefly, dogs exhibit brain atrophy, selective neuron loss, beta-amyloid plaques, oxidative brain damage, neuronal mitochondrial dysfunction, impaired neuronal glucose metabolism, and neuroinflammation due to abnormal microglial and astrocyte activity [17, 18]. Calcium regulation abnormalities have also been suggested [20]. Feline CDS is less understood but involves beta-amyloid plaques of distinct structure, neuronal loss, and brain atrophy [18]. Both canine and feline brains show the accumulation of several phosphorylated tau epitopes consistent with Alzheimer’s disease (AD) in humans [18], although they do not develop full-blown neurofibrillary tangles, possibly due to their shorter lifespans [21]. These similarities have positioned dogs [22] and cats [23] as potential models for studying AD in humans.

The role of nutrition in preserving cognition in pets and humans has been studied for decades, showing promising results in dogs and cats [24, 25]. These interventions are often perceived as “natural” by the public, aligning with owner preferences [26]. However, studies evaluating enriched diets and nutraceuticals in improving cognitive function in pets have reported inconsistent results [27], leaving no consensus on effective interventions.

This work aims to systematically review published studies assessing the efficacy of enriched diets and nutraceuticals in improving cognitive function in aging companion animals. The objectives are to identify relevant products and propose recommendations for future research in this field.

## Materials and methods

This review follows the Preferred Reporting Items for Systematic reviews and Meta-analyses (PRISMA) statement [28] and is registered at PROSPERO with registration number CRD42023451061. Since this study did not involve animals or humans, no ethical protocol was required.

### *Literature search*

The literature search was conducted on July 17th, 2023, across four databases: Pubmed (Medline, 1946 onwards), Web of Science (Clarivate, 1950 onwards), Dimensions (Digital Science, 1950 onwards), and CAB Abstracts (EBSCOhost, 1973 onwards). A specifically designed formula composed of three groups of keywords, (1) age-related cognitive dysfunction, (2) targeted species, and (3) enriched diets and nutraceuticals, was applied on titles and abstracts of the registered publications (Table 1). The third keywords group (enriched diets and nutraceuticals) was adapted from a systematic review focusing on enriched diets and nutraceuticals in the context of osteoarthritis pain management in dogs and cats [29]. A citation search was then performed on the identified articles, and relevant references were added.

**Table 1:** Categorization of keywords used in the search formula for identifying relevant studies

Keyword group	Keywords
1. Age-related cognitive dysfunction	memory, cognit*, learning, anxiety, “Cognitive dysfunction,” Alzheimer, senility, dementia
2. Targeted species	cat, cats, feline, dog, dogs, canine
3. Enriched diets and nutraceuticals^a^	“disease modifying agent,” nutrient*, nutritional, “nutritional medicinal product,” “nutritional supplements,” nutraceutical*, “botanical drugs,” “botanical food supplements,” “herbal health nutritionals,” “herbal health nutritional,” “herbal medicine,” “fortified food,” “food additive,” “food additives,” diet, “dietary supplements,” “dietary supplement,” dietary, “geriatric diet,” “natural product,” “natural products,” phytotherapy, “complementary medicines,” “complementary medicine,” homeopathy, antioxidant, “food-derived products,” “food-derived product,” “mineral supplements,” “mineral supplement,” supplement, supplements, “Medium chain triglycerides,” MCT, “Fatty acids”

Keywords within each group were connected by “OR.” The three keyword groups were combined using “AND.”

^a^Adapted from Barbeau et al.

All references, including those from the database search and citation search, were imported into Covidence (Veritas Health Innovation, Melbourne, Australia), a web-based collaboration software platform that streamlines the production of systematic and other literature reviews.

An updated literature search was performed on 19th November 2024, using the same databases and search formula. However, no new eligible articles were identified.

### Selection of studies

Covidence software automatically removed duplicates. Then, the entire pool of studies was independently screened by two reviewers (T.B., J.E.) in a two-step process. In the first step, titles and abstracts were evaluated based on the following inclusion criteria:

(I) The study was a clinical trial.
(II) The study was written in English.
(III) The study investigated the effects of enriched diets or nutraceuticals on the cognitive functions of aged cats or dogs.

Studies were excluded if they met any of the following criteria:

(I) The study did not include senior pets.
(II) No cognitive questionnaire or task was used.
(III) Enriched diets or nutraceuticals were used solely as adjunctive therapy to a drug.
(IV) Only an abstract was available.

In the second step, full texts of the selected studies were independently assessed for eligibility by the same two reviewers. Any disagreements during the screening or assessment process were resolved through thorough discussions between the reviewers (T.B., J.E.).

### Data extraction

Data extraction was conducted independently across all studies by two reviewers (T.B., J.E.), with the extracted data subsequently consolidated by T.B. to produce the final dataset using Excel (Excel 2023, v. 2310, Microsoft Corp.). The extracted information encompassed key general details, including publication date, journal, title, first author’s name, and the study’s country of origin. Pertaining to the animals involved, the extracted data covered number of animals, species, breeds, population specifications (quantity, sex ratio), and the source of the animals (e.g., laboratory, kennel, owners), alongside inclusion and exclusion criteria. Regarding dietary aspects, details on the diet at baseline (T0) and the control diet as well as the diet maintained during the study were extracted. The nature of the intervention (i.e., enriched diet or supplement) was specified, along with comprehensive information about its composition, dosage, frequency, and duration. Cognitive assessment details were also extracted, including the type of cognitive evaluation employed (cognitive tasks or questionnaires), the specific name of the assessment used, and the outcome measured.

### Quality assessment

The quality of studies was assessed independently by two reviewers (T.B., J.E.) using a modified CAMARADES checklist (Table 2) [29–31]. Based on the quality score (QS) distribution, studies scoring 18 or above were classified as very high quality, those scoring 16 and 17 were considered good quality, those scoring 14 and 15 were categorized as medium quality, and studies scoring less than 14 were classified as low quality.

**Table 2:** Modified CAMARADES checklist for study quality assessment

Item	Score
Publication in peer-reviewed journal	Yes (1), no (0)
Type of study	Single cohort (0), crossover (1), parallel (2)
Controlled study	No control group (0), positive control group (1*), placebo (1*)
Baseline testing	Yes, of everything studied (2), yes, but only part of things studied (1), no or not mentioned (0)
Randomization of treatment or control	Yes (1), no or not mentioned (0)
Blinding	Single-blinded (1), double-blinded (2), no or not mentioned (0)
Blinded assessment of outcome	Yes (1), no or not mentioned (0)
Prespecified inclusion and exclusion criteria	Yes (1), no (0)
Ethics committee approval indicated	Yes (1), no (0)
Animals check-up at baseline	Yes (1), no or not mentioned (0)
Sample size calculation	Yes (1), no or not mentioned (0)
Sample size	< 10 per group (0), 10–20 per group (1), > 20 per group (3)
Statement of providing diet fulfilling animals’ needs	Yes, and provides name or full composition (2), yes (1), no or not mentioned (− 1), no uniformization of diet (− 2)
Dose of enrichment or nutraceutical provided	Yes (1), no (− 1)
Cognitive assessment	Questionnaire made for the study (0), validated questionnaire (1*), task made for the study (0), validated task (1*)
Statement regarding possible conflict of interest	Yes (1), no (0)
Reporting animals excluded from analysis	Yes (1), no (0)
Reporting study funding	Yes (1), no (0)
Statistical analyses clearly described	Yes (1), no (0)

The normality of the quality scores was assessed by visualization of the density plot. Potential factors influencing the quality scores, the method of cognitive function assessment (questionnaire vs. cognitive task), type of intervention (enriched diet vs. supplementation), and the type of animals included (laboratory vs. owner-owned dogs), were evaluated using the Wilcoxon test. A *p*-value < 0.05 was considered statistically significant, while a *p*-value < 0.1 was regarded as a tendency warranting further investigation.

### Evaluation of cognitive function

The clinical trials were categorized separately based on whether they assessed cognitive functions through cognitive tasks or questionnaires. The numerous and diverse cognitive tasks used were grouped according to the cognitive function they assessed (i.e., memory, learning, executive function, and visuospatial function) [32, 33] (Table 3). One clinical trial employed the Modified Canine Cognitive Vienna Battery, consisting of 11 subtests. Due to its comprehensive evaluation of canine cognition, this test evaluates the four cognitive functions.

**Table 3:** Cognitive tasks used in the clinical trials and the cognitive function tested, task classification adapted from Davis and Head [32] and Martin et al. [33]

Cognitive function	Task	Number of uses Dogs | cats	References Dogs | cats
Learning	Object discrimination learning	7 | 1	[20, 34–38] | [35]
	Landmark task—land-0 only	6 | 0	[38–43]
	Size discrimination learning	3 | 1	[39, 40, 44–47] | [48]
	Black and white discrimination learning	3 | 0	[38, 39, 45]
	Maze learning	2 | 0	[49, 50]
	Spatial discrimination learning	1 | 0	[51]
Memory	Delayed non-matching position	9 | 1	[20, 34, 35, 37, 38, 40, 41, 44, 51, 52] | [48]
	Object discrimination retention	1 | 0	[37]
	Maze retention	1 | 0	[49]
Executive function	Attention task/oddity discrimination	5 | 0	[20, 40, 51, 53]
	Object discrimination reversal	2 | 1	[35, 51] | [35]
	Size discrimination reversal	2 | 1	[39, 40] | [48]
	Egocentric task reversal	2 | 0	[42, 43]
	Maze reversal	2 | 0	[49, 50]
	Black and white discrimination reversal	2 | 0	[38, 39]
	Spatial discrimination reversal	1 | 0	[51]
Visuospatial function	Landmark task	6 | 1	[38–43] | [48]
	Egocentric task	2 | 1	[42, 43] | [48]

### Supplementation

Regarding dietary supplementation, given that clinical trials often involve a combination of nutrients, a variable was created to categorize each existing nutrient: e.g., plant extracts and products, antioxidant vitamins, omega-3 fatty acids, apoaequorin, medium-chain triglycerides (MCT), coenzyme Q10 (CoQ10), B vitamins mitochondrial co-factors: lipoic acid (LA) without acetyl-L-carnitine (ALCAR), ALCAR without LA, or a combination (LA + ALCAR), tryptophan, phosphatidylserine (PS), and others: n-acetyl cysteine (NAC), s-adenosyl-methionine (SAMe), homotaurine, arginine.

To standardize dosages, all doses per kilogram of body weight for dogs were calculated based on a theoretical 10 kg dog, reflecting the average weight of laboratory beagles used in most studies. For cats, doses were calculated using a 4 kg baseline, as indicated in Pan et al.’s article [48]. When enriched diets were used, metabolizable energy (ME) and energy requirements were calculated according to National Research Council’s 2006 guidelines (130*BW^0.75 kcal/day for dogs and 100*BW^0.67 for cats) [54]. The theoretical food quantity was then derived by dividing energy requirements by the ME of the diet, allowing for the calculation of nutrient intake per animal.

## Results

### Study selection process

**Fig. 1:**
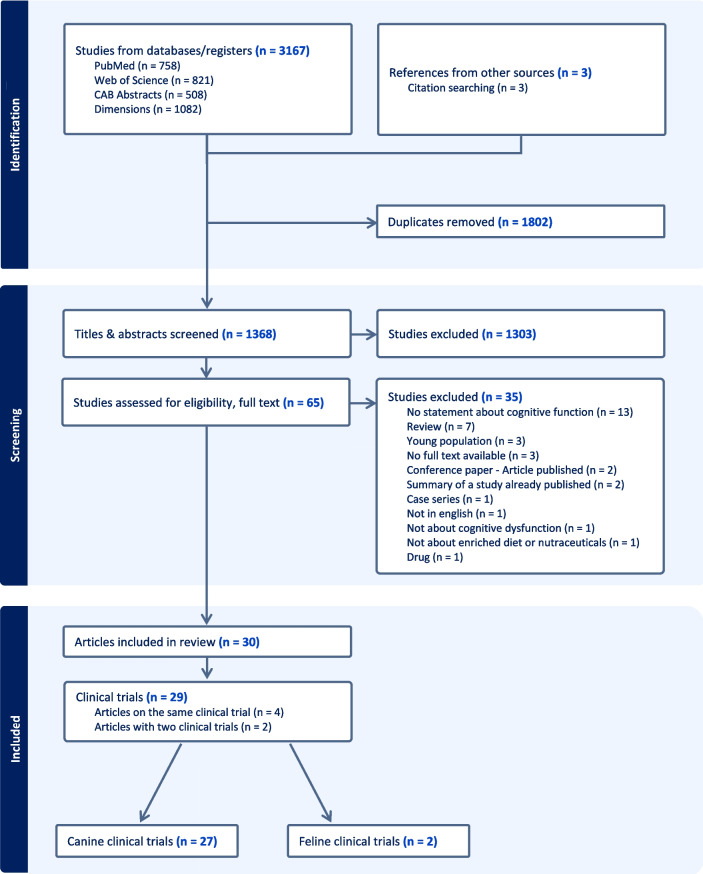
PRISMA flowchart for study selection

A total of 3167 articles were imported from databases and an additional 3 articles were sourced through citation searching, into Covidence software. The PRISMA flowchart is shown in Fig. 1. Covidence removed 1802 duplicates, leaving 1368 studies for title and abstract screening, which resulted in the exclusion of 1303 articles. Full texts of the remaining 65 studies were assessed for eligibility, leading to the exclusion of 35 studies. The main reasons for exclusion were as follows: no mention of cognitive function (37%), the study being a literature review (20%), inclusion of only young pets (9%), and unavailability of full text (9%). Additionally, four excluded articles were conference papers or summaries of previously published studies already included in the review. As a result, 30 studies were included, comprising 29 unique clinical trials. Notably, four articles referred to the same clinical trial [44–47], while two articles encompassed two distinct clinical trials each [20, 35]. Among these, only two clinical trials focused on cats [35, 48], with the remaining 27 trials centered on dogs.

### Characteristics of the studies

Studies were published between 2002 and 2023, the most prolific year being 2007 with four publications. The median number of authors per study was 6 (range 3–13), involving a total of 123 unique authors who contributed to these 30 studies, resulting in 174 author appearances. Of the 123 authors, 18 (14.6%) were affiliated with the University of Toronto, 13 (10.6%) with CanCog Technologies, and 11 (8.9%) with the University of California, Irvine. The corresponding authors were affiliated with institutions in the following countries: the USA (36.7%), Canada (26.7%), Italy (10.0%), Austria (6.7%), France (6.7%), and Germany, South Korea, Spain, and the UK (3.3% each).

### Clinical trial methodologies

Concerning the dog trials selected, 15 clinical trials were conducted on laboratory beagles; 11 were on pet dogs, which included multiple breeds and crossbred dogs; and one was on kennel dogs. All trials included a control group except four studies on pet dogs. The median number of dogs included in the test groups was 11.5 (range 4–61), with eight trials (30%) having less than 10 dogs and two trials (7%) having more than 40 dogs. Statistical power calculations were provided in three trials (11%). Six trials (22%) did not provide sufficient information to calculate the sex ratio. Of the remaining trials, four (15%) had a balanced distribution between males and females. Across all studies, the mean age of the dogs was 10.1 years (range 6.5–17.3). Two clinical trials did not provide precise age data for the dogs, only noting that they were “aged.” Data on dogs’ weights were provided in seven trials (26%). In 17 trials (63%), all dogs were fed a controlled complete and balanced food; in seven trials (26%), dogs were kept on their owners’ diets, which were not detailed in the articles; and two trials (7%) did not mention the dogs’ basal diet.

The two feline clinical trials involved laboratory domestic short-haired cats, included control groups, but did not provide sample size calculations nor information to calculate sex ratio. One trial involved 8 cats, age ranged 8.4 to 13.9 years old, and the other one involved 16 cats, age ranged 5.5 to 8.7 years old.

### Quality scores

**Fig. 2:**
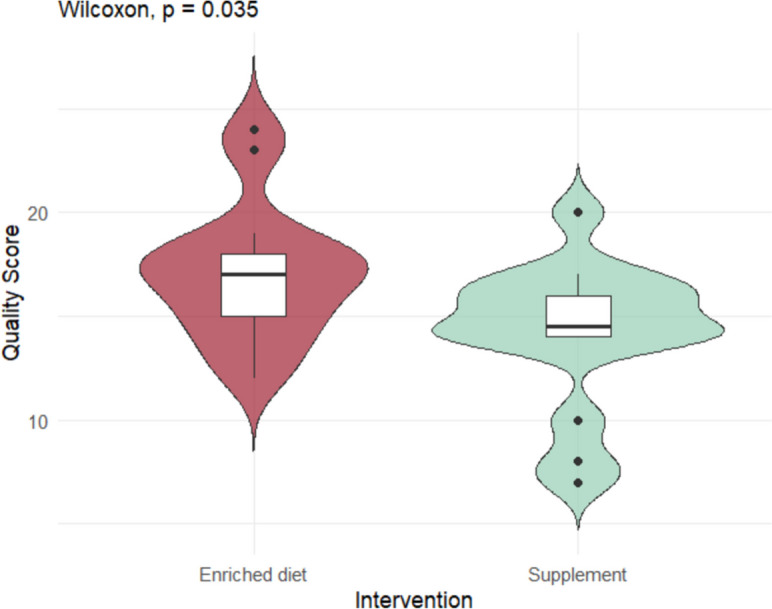
Quality score of the studies according to the intervention (enriched diet of supplementation)

**Fig. 3:**
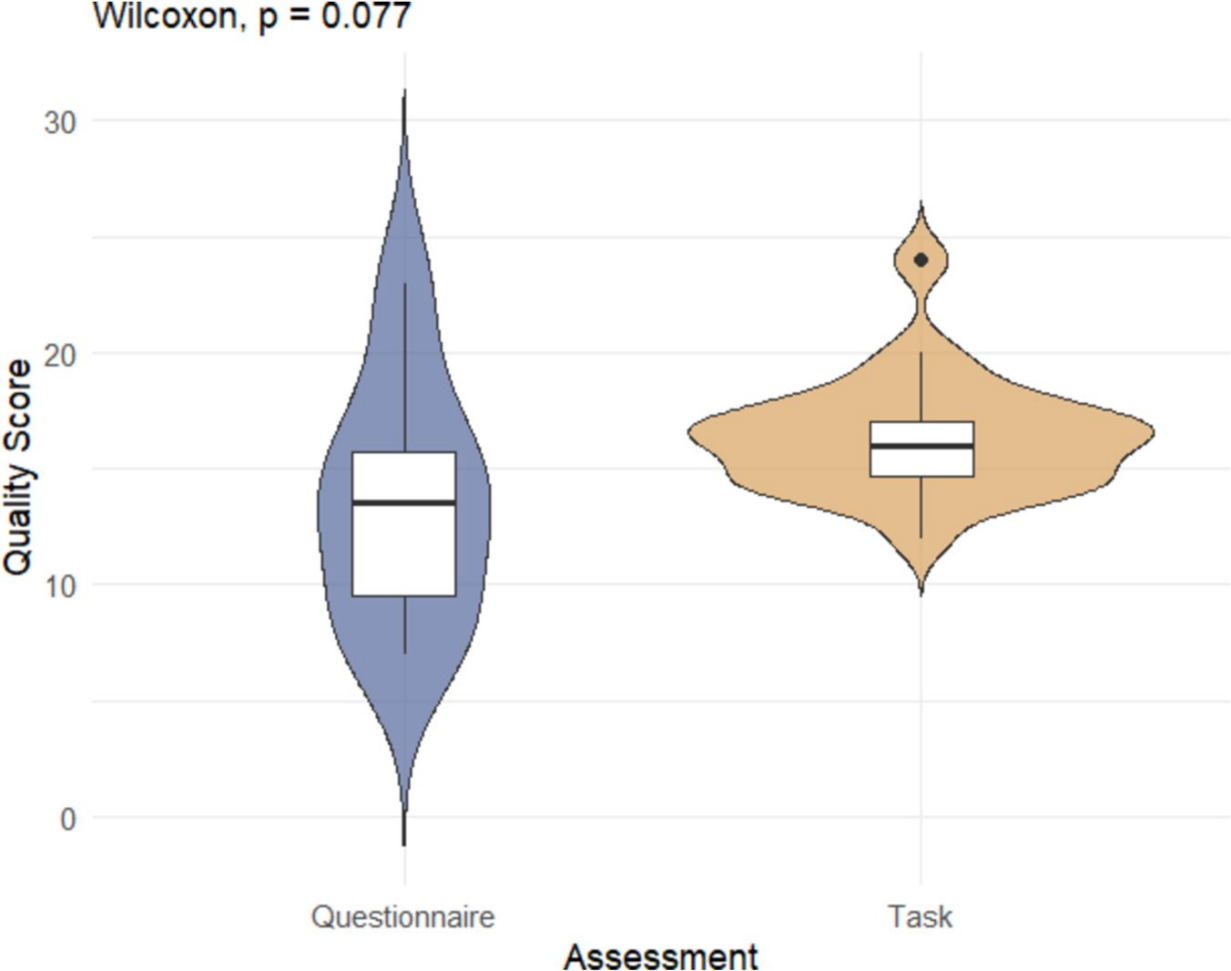
Quality score of the studies according to cognitive function assessment (cognitive tasks or questionnaire)

**Fig. 4:**
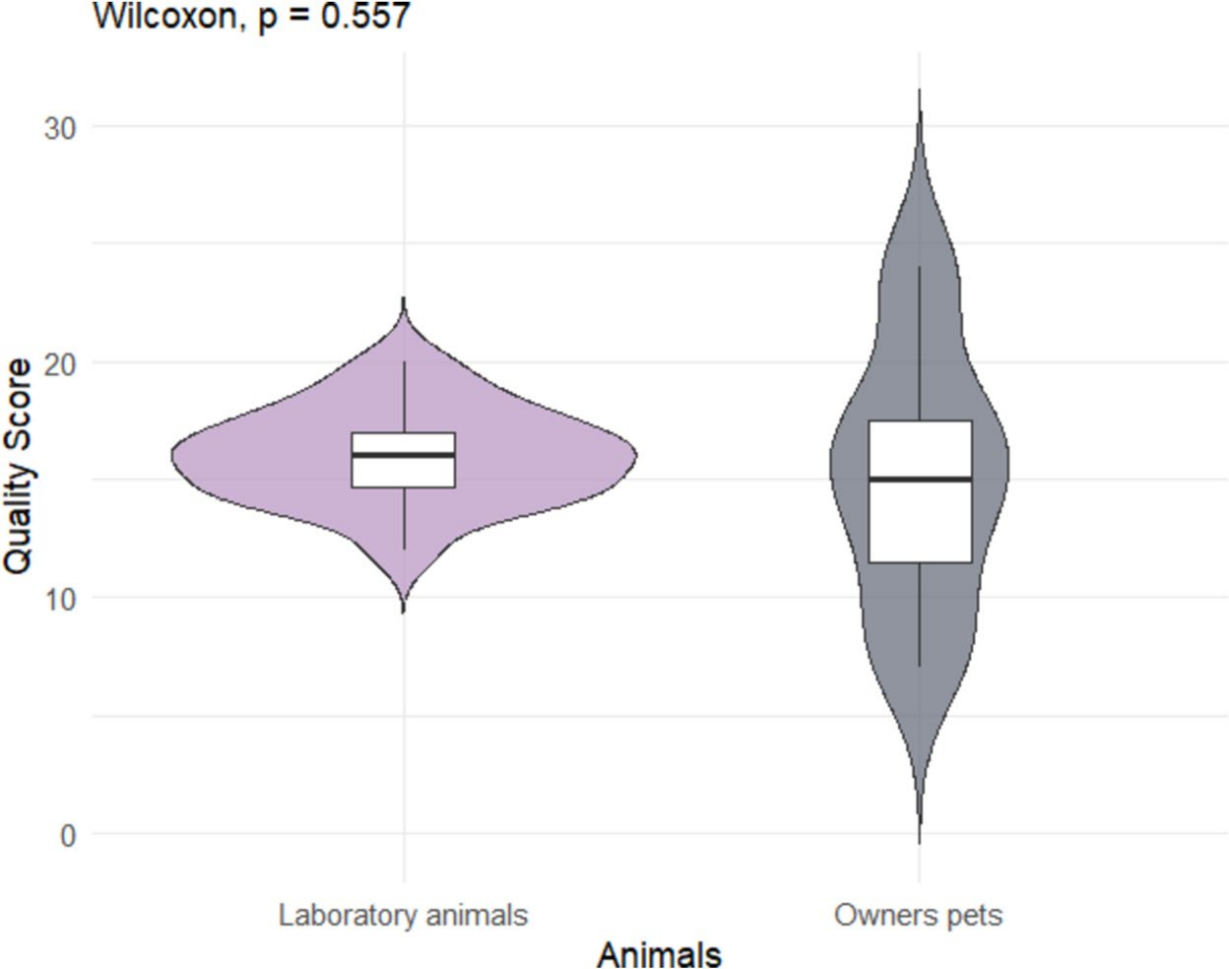
Quality score of the studies according to the animals included (laboratory animals or owner pets)

The quality scores of the studies, evaluated using the modified CAMARADES checklist, were not normally distributed. The median quality score was 16 (range 7–24). Due to the limited number of studies involving cats, factors influencing quality scores could only be analyzed for dogs. Studies using enriched diets had significantly higher quality scores than those using supplements (*p* = 0.035; median: 17.0 vs. 14.2, respectively; Fig. 2). While not statistically significant, studies using cognitive tasks tended to have higher quality scores compared to those using questionnaires (*p* = 0.077), with greater variability observed in the latter group (Fig. 3). Similarly, although no statistically significant difference was found in quality scores between studies involving laboratory dogs and owner-owned dogs, the latter group exhibited more variability in scores (Fig. 4).

### Cognitive evaluation

**Fig. 5:**
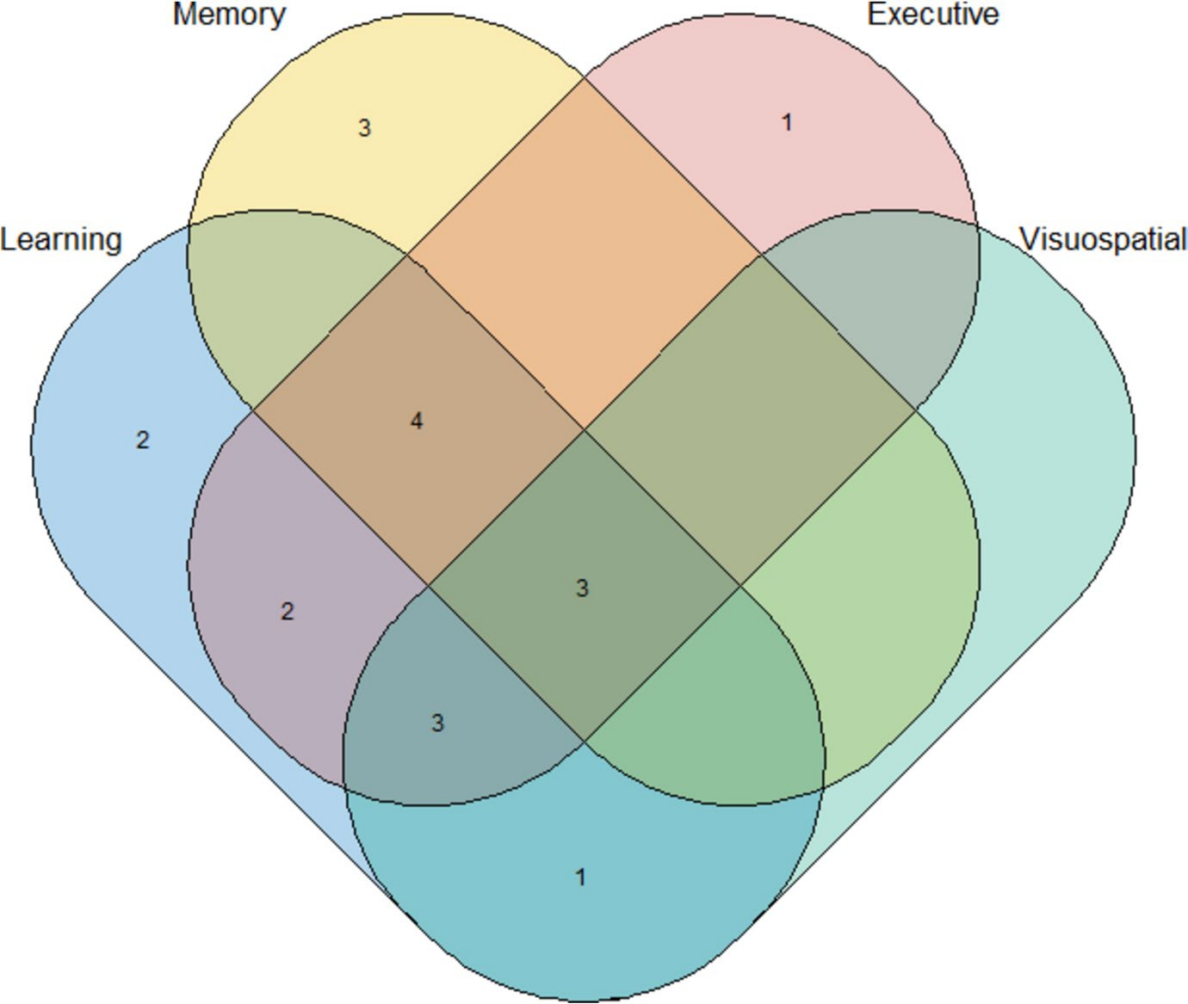
Venn diagram of the cognitive functions evaluated in 27 canine clinical trials

Of the 27 canine clinical trials reviewed, 19 trials (70%) incorporated cognitive tasks (Table 4) and eight (30%) used questionnaires (Table 5) for cognitive function evaluation. The most evaluated function was learning (*n* = 15), followed by executive function (*n* = 13), memory (*n* = 10), and visuospatial function (*n* = 7) (Fig. 5).

**Table 4:** Results of supplementation on cognitive functions

								Results on cognitive functions			
Trial	Number	Study population	Age (years)	Study design	Intervention	Duration (days)	Quality^a^	Learning	Memory	Executive	Visuospatial
Dogs											
[51]	24^b^	lab. beagle	6.5–13.9 Mean 8.6	Parallel controlled (12 CT/12 test)	Pork brain sphingolipids, DHA, EPA	168	Very high	Effect	No decline	Effect	Not tested
[37]	24	lab. beagle	8.6–11.1 Mean 9.8	Parallel controlled (12 CT/12 test)	DHA	175	Good	Effect	No effect	Not tested	Not tested
[43]	24^b^	lab. beagle	9.1–11.5	Parallel controlled (12 CT/12 test)	Fish oil, vitamins E and C, B vitamins, and arginine	191	Good	No effect	Not tested	Effect	Effect
[53]	23	lab. beagle	8.5–12.5	Parallel controlled (11 CT/12 test)	Vitamins E and C, L-carnitine, LA, fruits and vegetables^c^	275	Good	Not tested	Not tested	Effect	Not tested
[20]	24	lab. beagle	9.5–17.3	Parallel controlled (8 CT/8 low dose/8 high dose)	Apoaequorin	32	Good	Effect	No effect	Effect	Not tested
[42]	24	lab. beagle	7.5–11.6 Mean 9.8	Parallel controlled (12 CT/12 test)	MCTs	225	Good	No effect	Not tested	Effect	Effect
[50]	20	Pet dogs	10–17 Mean 13.4	Parallel controlled (9 CT/11 test)	Krill oil and powder, glucosamine sulfate, *Trametes versicolor, Boswellia serrata, Harpagophytum procumbens, Ginkgo biloba* extract, CoQ10, vitamin E	50	Good	Effect	Not tested	No effect	Not tested
[35]	14	lab. beagle	9.2–12.8	Parallel controlled (7 CT/7 test)	SAMe	55	Good	No effect	No effect	Effect	Not tested
[44–47]	24	lab. beagle	8.1–12.0	Parallel controlled (12 CT/12 test)	Vitamins E and C, L-carnitine, LA, fruits and vegetables^c^	1095	Good	Effect	Not tested	Not tested	Not tested
[20]	24	lab. beagle	10.2–15.9	Parallel positive CT (8 CT/8 low dose/8 high dose)	Apoaequorin	32	Good	Effect	Not tested	Effect	Not tested
[38]	37	lab. beagle	6.8–8	Parallel controlled^d^ (9 CT/9 test) (9 CT/9 test) (9 CT/9 test) (9 CT/10 test)	LA and ALCAR Vitamins E and C, fruits and vegetables^c^ LA, ALCAR, vitamins E and C, fruits and vegetables^c^ LA	1095	Medium	No effect No effect No effect Negative effect	Effect No effect No effect No effect	No effect No effect No effect No effect	No effect No effect No effect No effect
[49]	23	Kennel dogs	10–16	Parallel controlled (12 CT/11 test)	Homotaurine	731	Medium	Effect	No decline	Effect	Not tested
[40]	13	lab. beagle	8.2–9.6 Mean 8.6	Parallel controlled (6 CT/7 test)	Green tea and *Piper nigrum* extracts, curcumin, LA, N-acetyl-l-cysteine	275	Medium	No effect	No effect	No effect	Effect
[41]	12	lab. beagle	7.6–8.8 Mean 8.2	Parallel controlled (6 CT/6 test)	LA and ALCAR	76	Medium	Effect	Not tested	Not tested	Effect
[34]	9	lab. beagle	7–12.7 Mean 8.2	Crossover	*Ginkgo biloba* extract, vitamin E, PS, vitamin B6	70*2	Medium	Not tested	Improvement	Not tested	Not tested
[55]	94	Pet dogs	6.1–14 Mean 9.1	Parallel controlled (49 CT/45 test)	Green tea polyphenols, vitamins E and C, DHA, PS, tryptophan	365	Very high	No effect	No effect	No effect	No effect
[52]	35	lab. beagle	8–14.5 Mean 10	Parallel controlled (11 CT/12 low dose/12 high dose)	Polyphenol-rich extract from grape and blueberry	75	Good	Not tested	No effect	Not tested	Not tested
[36]	79	Pet dogs	7.1–14.4 Mean 9.73	Parallel controlled (41 CT/38 test)	Vitamins E and C, polyphenols, DHA, PS, tryptophan	365	Good	No effect	Not tested	Not tested	Not tested
[39]	30	lab. beagle	7.8–11.2	Parallel controlled^e^ (10 CT/10 test) (10 CT/10 test) (10 CT/20 test)	LA ALCAR LA and ALCAR	129 129 79	Good	No effect No effect No effect	Not tested Not tested Not tested	No effect No effect No effect	No effect No effect No effect
Cats											
[48]	32	lab. DSH	5.5–8.7 Mean 6.7	Parallel controlled (16 CT/16 test)	Fish oil, vitamins E and C, B vitamins, and arginine	345	Good	Effect	Effect	Effect	Effect
[35]	16	lab. various breeds	8.4–13.9	Parallel controlled (8 CT/8 test)	SAMe	54	Good	No effect	Not tested	Effect	Not tested

^a^Quality: based on the quality score from the adapted CAMARADES checklist, studies scoring 18 or above were classified as very high quality, those scoring 16 and 17 were considered good quality, those scoring 14 and 15 were categorized as medium quality, and studies scoring less than 14 were classified as low quality

^b^Sample size calculation provided

^c^Spinach flakes, tomato pomace, grape pomace, carrot granules, and citrus pulp

^d^The 9 dogs in the CT groups are the same, vs 4 supplemented groups of 9 or 10 dogs

^e^During the first 129 days, the dogs were divided into three groups: CT (*n* = 10), ALCAR (*n* = 10), and LA (*n* = 10). After this period, the ALCAR and LA groups were combined, resulting in two groups for the next 79 days: the CT group (*n* = 10, unchanged) and the ALCAR + LA group (*n* = 20), which included the dogs from both the ALCAR and LA groups

*lab.* laboratory, *DSH* domestic shorthair, *CT* control group, *ALCAR* acetyl-L-carnitine, *DHA* docosahexaenoic acid, *EPA* eicosapentaenoic acid, *LA* lipoic acid, *MCTs* medium-chain triglycerides, *PS* phosphatidylserine, *SAMe* s-adenosyl-methionine.

**Table 5:** Results of supplementation on cognitive questionnaires, only canine clinical trials

Trial	Number	Study population	Age (years)	Study design	Intervention	Quality^a^	Duration (days)	Questionnaire	Result
[56]	125	Pet dogs	7–20 Mean 12	Parallel controlled (64 CT/61 test)	Vitamins E and C, fruits and vegetables, LA, ALCAR, omega-3 fatty acids	Good	60	Custom-made	Effect: interaction, activity, compulsive behaviors No effect: disorientation, sleep–wake cycle, house training
[57]	36	Pet dogs	8–17 Mean 11.5	Parallel controlled (19 CT/17 test)	SAMe	Medium	60	Custom-made	Effect on global score
[58]	27	Pet dogs	> 8	Parallel controlled (16 CT/11 test)	Vitamins E and C, DHA, EPA, PS, LA, ALCAR, L-carnitine, CoQ10, selenium, NAC	Medium	42	Custom-made	Effect: disorientation, interaction, sleep patterns, housesoiling
[59]	78^b^	Pet dogs	9–16	Parallel controlled (29 CT/26 test)	Vitamins E and C, DHA, EPA, B vitamins, arginine and MCTs	Very high	90	DISHAA^c^	Improvement: disorientation, social interaction, anxiety, sleep–wake cycle, house training, learning and memory, activity
[60]	9	Pet dogs	7.0–14.4 Mean 10.3	Longitudinal uncontrolled	Cyanidin-3-glucoside from honeyberry	Medium	90	DISHAA^c^	Improvement on global score
[61]	37	pet dogs	Mean 11.4	Longitudinal uncontrolled	*Gingko biloba* leaf extracts	Low	56	Custom-made	Improvement: disorientation, sleep patterns, behavioral changes, general behavior, general physical condition No effect: house training
[62]	10	Pet dogs	9–17 Mean 13	Longitudinal uncontrolled	Curcumin, *Salvia miltiorrhizae, Polygala tenuifolia*, vitamin E, PS, CoQ10, SAMe, zinc	Low	90	DISHAA^c^	Improvement on global score
[63]	8	Pet dogs	> 7	Longitudinal uncontrolled	*Ginkgo biloba* extracts, vitamin E, PS, and vitamin B6	Low	90	Adapted from other publications	Improvement: socio-environmental interaction, disorientation, sleep–wake cycles, housesoiling, general activity

^a^Quality: based on the quality score from the adapted CAMARADES checklist. Studies scoring 18 or above were classified as very high quality, those scoring 16 and 17 were considered good quality, those scoring 14 and 15 were categorized as medium quality, and studies scoring less than 14 were classified as low quality

^b^Sample size calculation provided

^c^The DISHAA questionnaire, originally introduced by Pan et al. in 2018, is based on six categories (DISHAA) and incorporates questions from previously validated questionnaires. This tool was subsequently used in studies by Lee et al. (2022) and Dewey et al. (2023)

*CT* control group, *ALCAR* acetyl-L-carnitine, *DHA* docosahexaenoic acid, *EPA* eicosapentaenoic acid, *LA* lipoic acid, *MCTs* medium-chain triglycerides, *PS* phosphatidylserine, *SAMe* s-adenosyl-methionine.

Among the questionnaires, four were custom-designed based on observed symptoms of canine cognitive dysfunction, and two were adaptations of previously published questionnaires, including Pan et al.’s questionnaire [59], which was subsequently used in two other studies. Only one study employed both a questionnaire (specifically, the canine cognitive dysfunction rating scale, CCDR [64]) and a cognitive task [55]. However, the questionnaire was used solely for group equilibration purposes.

The two feline trials exclusively employed cognitive tasks, with no use of questionnaires. Both trials assessed learning and executive function, while one also evaluated memory and visuospatial function.

Specific tasks were commonly employed to assess various cognitive domains: Object Discrimination Learning Task emerged as the preferred method for evaluating learning, the Delayed Non-Matching Position Task for memory assessment, the Attention Task/Oddity Discrimination Task for executive function evaluation, and the Landmark Task for assessing visuospatial function (Table 3).

### Supplementation

In dogs, 15 trials (56%) tested a supplement, while 12 trials (44%) evaluated an enriched diet. The median duration of treatment was 91 days, with a range of 32 days to 3 years. For cats, one trial tested a supplement lasting 39 days, and another tested an enriched diet lasting nearly a year (345 days). For 89% of the dog studies and all the cat studies, the precise dosage administered to each animal was provided.

The most frequently used nutrients were plant extracts and products (*n* = 14, 48%), and vitamins E and/or C (*n* = 14, 48%). These were followed by omega-3 fatty acids (*n* = 10, 34%), mitochondrial cofactors (LA, ALCAR, or a combination, *n* = 8, 28%), and phosphatidylserine (*n* = 6, 21%). The 29 clinical trials and their results are summarized in Table 4 for trials that included task-based assessments and in Table 5 for those that used questionnaires.

In trials testing eicosapentaenoic acid (EPA) and docosahexaenoic acid (DHA), dosages ranged from 7 to 94.5 mg/kg. For vitamin E, dosages varied between 1.2 and 19 mg/kg, while those including LA ranged from 2 to 11 mg/kg. ALCAR dosages spanned 1 to 27.5 mg/kg, and phosphatidylserine ranged from 0.2 to 6.3 mg/kg. Detailed dosage information for each supplement in the studies can be found in Supplementary File 1.

### Effects of the supplements on cognitive functions

#### Supplements that consistently failed to show positive effects

Tryptophan was tested in two canine trials (86 mg/kg in one, unknown dosage in the other) and consistently failed to demonstrate a positive effect on cognitive functions [36, 55].

#### Supplements that showed a positive effect by their own

- Omega-3 fatty acids: In one trial (QS: 19), DHA alone (approx. 26 mg/kg) improved learning but did not affect memory [37]. A second trial (QS: 20) combining pork brain sphingolipids with 67.5 mg/kg of DHA and 27 mg/kg of EPA showed benefits for learning and executive functions and prevented memory decline [51].
- MCT: One trial (QS: 17) demonstrated that a diet containing 5.5% MCTs significantly improved executive and visuospatial functions in laboratory dogs [42].
- S-adenosyl-methionine: In trials involving cats (QS: 15) and dogs (QS: 16), SAMe supplementation improved executive functions but had no effect on learning. In cats, this benefit was observed only in the top performers [35]. A 2-month trial in pet dogs (QS: 15) also showed SAMe significantly alleviated CCD symptoms [57].
- Homotaurine: A 1-year trial (QS: 14) found that homotaurine significantly improved learning and executive functions and prevented age-related memory decline in dogs [49].
- Apoaequorin: In a 32-day trial (QS: 17), apoaequorin improved learning and executive functions without affecting memory in dogs [20]. A higher dose resulted in better cognitive outcomes, and a second trial (QS: 16) showed greater performance compared to selegiline.

#### Supplements effective only in combination

##### Plant extracts and products, vitamins E and C

Plant extracts and products and antioxidant vitamins showed limited efficacy when used alone. For instance, polyphenols from grape and blueberry extracts (QS: 18) did not significantly improve memory in dogs after 75 days [52]. Similarly, Snigdha et al. (QS: 15) found no significant effects of a fruit and vegetable and antioxidant vitamin–enriched diet on learning, memory, executive, or visuospatial functions in beagle dogs over 3 years [38]. However, combinations of the same fruits and vegetables with antioxidant vitamins, LA, and L-carnitine were effective in two trials [46, 53]. Additional combinations with omega-3 fatty acids were also beneficial in both dogs [50, 56] and cats [48].

##### LA and ALCAR

LA alone, tested at doses of 2.7 mg/kg and 11 mg/kg, failed to show benefits and even negatively impacted learning at the lower dose after 3 years of supplementation in dogs [38, 39]. Similarly, ALCAR, tested once at 27.5 mg/kg, also tended to worsen learning performance [39]. Despite the negative effects observed with LA and ALCAR when used individually, their combination demonstrated a positive effect on memory in dogs at doses of 2.7 mg/kg LA and 5.4 mg/kg ALCAR [38]. Christie et al. (2009) did not observe any positive effects at higher doses (11 mg/kg LA and 27.5 mg/kg ALCAR), while Milgram et al. (2007) reported improvements in learning and visuospatial functions using the same dosages. However, it is important to note that neither Christie et al. (2009) nor Milgram et al. (2007) tested memory function. Christie et al. proposed that differences in the cognitive baseline of the animals could explain the divergent results [39].

#### Supplements consistently effective but only used in combination

Certain supplements, such as arginine (315–390 mg/kg), NAC (4–17 mg/kg), L-carnitine (2.7–4.7 mg/kg), and B vitamins, consistently showed positive effects but were always used in combination with other nutrients. These supplements were absent from trials that failed to find positive effects (see Supplementary File 1 for dosages).

#### Ineffective combinations

- Omega-3 fatty acids + tryptophan: The only two trials that included omega-3 fatty acids and failed to show any positive effects were the ones combining omega-3 fatty acids with tryptophan [36, 55].

#### Effective combinations

- Omega-3 fatty acids + CoQ10: The two trials with the lowest effective doses of EPA and DHA (with 15 mg/kg of krill oil and 5 mg/kg of krill powder [50], and with 7 mg/kg of EPA + DHA [58]) both incorporated CoQ10 (1.5 mg/kg and 0.2 mg/kg, respectively).
- Omega-3 fatty acids in combination cocktails: One trial in laboratory dogs (QS: 18) [43] and one in laboratory cats (QS: 18) [48] tested enriched diets containing vitamins E and C, EPA, DHA, B vitamins, and arginine, both demonstrating positive effects on executive and visuospatial functions. The feline trial also showed improvements in learning and memory. A similar enriched diet, further supplemented with 5.5% or 9% MCTs, was tested in pet dogs and showed improvements across all DISHAA domains after 90 days (QS: 23) [59]. Two other trials demonstrated benefits for CCD symptoms in pet dogs with combinations including vitamins E and C, omega-3 fatty acids, and mitochondrial co-factors [56, 58].

## Discussion

This study aimed to identify, through a systematic review, effective enriched diets and nutraceuticals that could improve cognitive function in aging dogs and cats. We will first discuss the molecules that have been tested, and then finish by looking at the obstacles preventing us from going any further in our conclusions.

### Omega-3 fatty acids

DHA appears to have significant positive effects on the cognition of aging dogs and cats, whether administered alone or in combination with EPA. Clear positive results have been observed at high doses of DHA (33, 41, and 67.5 mg/kg) and EPA (37, 43, and 27 mg/kg) for both dogs and cats [43, 48, 51]. With lower doses, the effects are more variable [36, 55]. In dogs, these benefits are particularly evident in learning functions [43, 51], while in cats, positive effects have been noted across all studied cognitive functions when administered in combination [48]. Indeed, omega-3 fatty acids, especially EPA and DHA, play a crucial role in brain health. DHA, which is abundantly present in dogs and cats’ brain [65], is known to have neuroprotective effects. It improves synaptic membrane fluidity, reduces pro-inflammatory metabolites from omega-6 fatty acids, enhances antioxidant defenses, promotes neurogenesis, and increases glucose transporter activity [66]. The use of omega-3 fatty acids in humans leads to the maintenance of brain volume [67] and higher hemoglobin oxygen saturation and total hemoglobin concentrations, suggesting improved blood circulation in the brain [68]. In vitro, DHA needs to be protected with antioxidants to prevent its oxidation. Therefore, in vivo, while antioxidant defenses are generally sufficient in a physiological state, a combination of omega-3 fatty acids and antioxidants seems more reasonable [66].

Interestingly, the two studies that included coenzyme Q10 (CoQ10) alongside low doses of EPA and DHA reported positive effects on cognitive function [50, 58]. CoQ10, also known as ubiquinone or ubidecarenone, is a potent antioxidant that plays a critical role in cellular energy metabolism. In its reduced form, it is a key component of the mitochondrial electron transport chain facilitating the transport of electrons from Complex I and Complex II to Complex III [69]. Omega-3 fatty acids also contribute significantly to mitochondrial function by stabilizing complexes III and IV within this pathway [70]. This intricate interplay between omega-3 fatty acids and CoQ10 in the inner mitochondrial membrane suggests a synergistic relationship that supports mitochondrial efficiency [71]. Given that cognitive dysfunction is linked to oxidative stress and mitochondrial impairment, an intervention targeting mitochondrial metabolism may represent an effective strategy.

### Plant extracts and products, vitamins E and C

Studies on the administration of polyphenols or fruits and vegetables with vitamins E and C alone have failed to demonstrate significant efficacy on the cognitive function of dogs [38, 52]. This result was unexpected as one known cause of cerebral aging in dogs and cats is the accumulation of oxidative stress-related damage [18]. Indeed, antioxidant can inhibit the formation of excessive ROS and other free radicals, as well as bind metal ions that catalyze ROS generation [72]. The actual lack of efficacy of supplementing antioxidants alone is uncertain, just as the absence of a positive effect may be due to study design (treatment duration and dose, number of animals included, choice of cognitive assessment tools). Besides, antioxidants at high doses can act as pro-oxidants and also disrupt the redox balance by interacting with ROS at physiological concentrations necessary for optimal cellular function. These contradictory effects are referred to as the “antioxidant paradox” and have been previously reviewed [73]. For instance, research has shown that supplementing human diets with high doses of vitamin C (500 mg/day, approximately 6.7 mg/kg) increased oxidative damage in lymphocyte DNA. All the studies in this review were below this threshold, except for one that used 11 mg/kg of vitamin C, which failed to demonstrate any beneficial effects, possibly due to the high dosage [74]. Furthermore, the three studies using extracted polyphenols in dogs also showed no cognitive benefits [36, 52, 55] (Table 4). This aligns with the growing hypothesis that the health benefits linked to plant food consumption may not be attributable to individual compounds but rather to the synergistic actions of complex mixtures of phytochemicals and nutrients present in whole fruits and vegetables [75, 76].

Moreover, cognitive dysfunction is a multifaceted syndrome; therefore, while antioxidants may help mitigate oxidative damage, they may not be sufficient on their own. Supporting overall cerebral function may require a more comprehensive approach that includes additional therapeutic strategies beyond antioxidant supplementation.

When combined with mitochondrial cofactors, antioxidants may be beneficial for visuospatial function [40], executive function [53], and memory [44–47], but the results are inconsistent. Two studies showed positive effects [44–47, 53], while a third study, which included fewer dogs and may have lacked statistical power, did not demonstrate any effects with the same enriched diet [38]. Additionally, even if combining antioxidants with phosphatidylserine (which is known to protect certain neuronal populations from cell death [77]) has the potential to improve cognitive function, no positive effects have been yet demonstrated compared to a placebo. Antioxidants might also be beneficial when combined with EPA and DHA, though study outcomes appear to be dosage-dependent of these latter. A combination with omega-3 fatty acids and mitochondrial cofactors might be particularly interesting for pet dogs showing signs of CDS, but further research is needed, as only one good-quality trial has investigated this combination [56].

### Tryptophan and omega-3 fatty acids

Understanding the mechanisms behind tryptophan’s influence on cognition is essential for interpreting the clinical trials that failed to demonstrate its efficacy, particularly in conjunction with omega-3 fatty acids. Tryptophan’s role in cognitive function is complex, primarily due to its conversion into serotonin and its involvement in the kynurenine pathway, which produces both neuroprotective, antioxidant (kynurenic acid) and neurotoxic, pro-oxidative (quinolinic acid) metabolites [78]. Tryptophan competes with branched-chain amino acids for transport into the brain; in aging dogs, reduced physical activity could lead to decreased tryptophan uptake, limiting its availability for serotonin synthesis [79]. As a result, supplementation in low-activity dogs could increase the kynurenine pathway, potentially leading to higher levels of the pro-oxidative quinolinic acid. This, in turn, could oxidize omega-3 fatty acids, inhibiting their cognitive benefits. While omega-3 fatty acids are generally known to promote serotonin synthesis, they have also been associated with increased kynurenine levels, which may complicate their effects on cognition [80].

In the context of the two canine trials testing tryptophan, both studies failed to show positive cognitive effects, which may be due to several factors. First, the trials conducted by Chapagain et al. (2018, 2020) used a diet enriched solely with DHA. Second, one of the studies included high doses of vitamin C, potentially acting as a pro-oxidant as discussed above, while the other did not specify the dosage. Moreover, unless there is a deficiency, tryptophan supplementation may not yield significant improvements in executive function, as a balanced diet typically supplies adequate levels of this amino acid [79]. Additionally, only one of these studies evaluated the impact on executive function, which is the cognitive domain most likely to be influenced by tryptophan. This study involved pet dogs, where achieving statistical significance can be challenging due to high variability among individual responses.

### Acetyl-L-carnitine (ALCAR), L carnitine, and α-lipoic acid (LA)

ALCAR provides acetyl groups for acetylcholine production and supports mitochondrial fatty acid β-oxidation, contributing to neuroprotection by maintaining mitochondrial membrane integrity and promoting acetylcholine and glutathione (GSH) synthesis [81]. Although L-carnitine is essential for shuttling fatty acids into mitochondria for β-oxidation, it has not previously demonstrated the same oxidative stress benefits as ALCAR [82]. This difference is likely due to ALCAR’s superior ability to cross the blood–brain barrier. In the three clinical trials reviewed, L-carnitine was associated with positive effects but was always administered in combination with other compounds. In the only study using ALCAR alone (27.5 mg/kg), cognitive performance tended to be worse in the treated group. Much like excessive antioxidant use, high doses of ALCAR can increase oxidative stress [83]; therefore, this dosage may have been too high. Moreover, both ALCAR and L-carnitine appear more effective in energy-depleted conditions, such as aging or fatigue, rather than in healthy individuals [84].

Additionally, LA, a cofactor in mitochondrial energy production and acetylcholine synthesis, showed mixed outcomes [85]. While LA can reduce free radicals and inflammation, high doses may paradoxically act as pro-oxidants [86], which could explain why the two clinical trials using LA alone failed to show cognitive improvement, with one trial even reporting worsened outcomes. Due to the known sensitivity of cats to LA’s toxic effects [87], its use has not been tested in this species.

The combination of ALCAR and LA demonstrated improved cognitive outcomes, particularly in memory, in two out of three trials that tested this pairing alone, highlighting the potential synergistic effect between the two compounds.

### Medium chain triglycerides (MCTs)

In dogs, the cognitive benefits of MCTs have been demonstrated in two high-quality trials [42, 59]. A dietary inclusion of 5.5–6.5% MCTs (97% caprylic acid and 3% capric acid) over a 90-day period was sufficient to achieve these positive effects. While glucose is the brain’s primary energy source, its availability decreases with age due to mitochondrial dysfunction and reduced glucose metabolism [88]. In beagles, regional cerebral glucose metabolism has been shown to decline by as much as 25% by the age of six [89], contributing to age-related cognitive decline. MCTs offer an effective alternative energy source for the aging brain, as brain uptake of ketones remains unaffected in mild-to-moderate Alzheimer’s disease compared to healthy age-matched controls [90]. Unlike long-chain triglycerides, MCTs are rapidly digested without the need for pancreatic lipases or bile acids and quickly transported to the liver, where they are converted into ketones [91]. In addition to providing energy, MCTs and their derived ketones offer neuroprotective effects, such as reducing oxidative stress [92] and potentially inhibiting Aβ-induced glutamate release, which may decrease hyperexcitability and inflammation [93]. These findings suggest that incorporating MCTs into the diet not only compensates for energy deficits in aging brains but also provides broader cognitive support, positioning MCTs as a promising intervention for maintaining cognitive health in older dogs.

### S-adenosylmethionine (SAMe)

SAMe has shown positive results in three studies (one involving laboratory dogs, one with pet dogs, and one with laboratory cats) particularly in improving executive function in laboratory animals. SAMe plays a key role as the major methyl donor in the conversion of phosphatidylethanolamine (PE) to phosphatidylcholine (PC), a process that helps regulate mitochondrial membrane fluidity and integrity through the PC/PE ratio [94, 95]. It also serves as a precursor for cysteine, which is essential for the synthesis of GSH in neurons, further supporting its neuroprotective potential [96].

### Apoaequorin

Apoaequorin, a calcium-buffering protein, has demonstrated cognitive benefits in learning and executive functions in two clinical trials, likely by reducing excitotoxicity and preventing ischemic cell death associated to calcium dysregulation [97, 98].

### Homotaurine

One study investigated the effect of homotaurine alone on cognition on dogs and yielded positive results [49]. Homotaurine acts as a potent GABA_A_ receptor agonist [99], potentially reducing Aβ-induced excitotoxicity. It can also bind soluble Aβ peptides, interfering with the amyloid cascade [100], and its sulfur content may offer protection against oxidative damage [101].

While these findings on SAMe, apoaequorin and homotaurine are promising, more studies are needed to confirm their overall effectiveness in improving elderly dogs and cats’ cognitive functions.

### Other supplements

Other potentially promising supplements include arginine, NAC, and B vitamins, each consistently associated with positive effects but only used in combination with other compounds. Arginine serves as the sole precursor of nitric oxide, which is crucial for maintaining synaptic plasticity [102]. NAC acts as a precursor to GSH, a key antioxidant [103]. B vitamins are essential for energy production and amino acid metabolism, including homocysteine, which interacts with vascular and neuronal systems (for a review, see [104]).

### Limitations

There have been relatively few clinical trials conducted on dogs and even fewer on cats. The variability in protocols, particularly in cognitive assessment methods and the choice of supplements, has made it difficult to draw definitive conclusions. Moreover, supplements are often administered in combination to enhance nutrient effects and counteract the multi-faceted mechanisms of cognitive decline [72, 105]. This makes it challenging to draw conclusions about the efficacy and relevance of each individual nutrient. The absence of sample size calculations further limits the ability to determine whether a lack of effect is due to insufficient statistical power or a true absence of benefit [106]. Therefore, in cases where multiple cognitive tasks assessing the same function produce conflicting results, we opted to note an “effect,” as a non-effect in one task could result from inadequate statistical power or inappropriate task difficulty. Unfortunately, many studies on pet dogs lack control groups, standardized diets, and consistent feeding protocols before and during trials. This variability can influence the animals’ responses to supplements [38], as their nutritional status is often affected by incomplete and unbalanced feeding practices by owners [107].

Despite these limitations, this systematic review provides a comprehensive overview of clinical trials investigating supplements or enriched diets for improving cognitive function in dogs and cats, while also evaluating their quality. It highlights the barriers to obtaining definitive results, suggesting that future clinical trials should address these issues to enable future systematic reviews and ideally, meta-analyses.

### Recommendations for future trials and perspectives

Since dogs share their owners’ lifestyle factors such as physical activity, dietary choices, social relationships, and exposure to pollutants, they represent a compelling model for studying human aging [108, 109]. In contrast, laboratory dogs lack these human-like environmental influences, making it essential to test enriched diets and supplements in pet dogs, particularly in relation to cognition. Trials should ensure that the diet is standardized before the study begins and remains consistent throughout the trial period. Additionally, all studies should include a control group, especially when using subjective tools like questionnaires. The sample sizes of the groups should be determined based on statistical power calculations.

The use of established owner-administered questionnaires for cognitive function assessment in dogs, such as the DISHAA Assessment Tool [59, 110], CCDR [64], and Canine Dementia Scale [111], could help reduce disparities between studies. Questionnaires are useful for identifying behavioral deficits, while cognitive tasks offer an objective method to assess cognitive functions [112]. Therefore, the use of both methods is of great interest. Given the potential for nutrients to selectively affect specific cognitive functions [113], it is important to assess multiple cognitive functions using a variety of tasks to ensure that no effects are overlooked. To date, no study has used both questionnaires and cognitive tasks together in this context.

Additionally, evaluating the persistence of post-treatment effects would be valuable. Only one study has addressed this aspect, finding that while cognitive functions improved during a 50-day intervention, they returned to baseline levels within 10 days after the treatment ended [58].

## Conclusion

In conclusion, omega-3 fatty acids, particularly EPA and DHA, have shown significant cognitive benefits in aging dogs and cats, especially when administered at higher doses, suggesting their potential as effective interventions for cognitive decline. While antioxidants alone did not demonstrate clear efficacy, they remain essential in protecting omega-3 fatty acids from oxidation, ensuring their continued effectiveness. Other supplements, such as S-adenosyl methionine, medium-chain triglycerides, homotaurine, and apoaequorin, have also shown promising cognitive benefits in aging pets. However, to draw more definitive conclusions, future trials must standardize diets and feeding protocols, include control groups, and assess cognitive function using both objective tasks and subjective questionnaires. Moreover, careful calculation of sample sizes based on statistical power is crucial for producing reliable and meaningful results.

## Supplementary Information

Below is the link to the electronic supplementary material.

Supplementary file1 (DOCX 35 KB)

## Data Availability

The data that support the findings of this study are available from the corresponding author upon reasonable request.
