# Supplementary material for: Enhancing cognitive functions in aged dogs and cats: a systematic review of enriched diets and nutraceuticals
Source: GeroScience. 2025 Jan 18;47(3):2925–47. doi: 10.1007/s11357-025-01521-z (PMC12181554; doi:10.1007/s11357-025-01521-z)
Supplement: Supplementary file 1 — Supplementary file1 (DOCX 35 KB) [file 11357_2025_1521_MOESM1_ESM.docx]

| **Trial ID** | **Effect** | **QS** | **Duration (d)** | **Plant extracts and products** | **Vit. E / C** | **Omega-3 FA** | **Mitochondrial cofactors** | **PS** | **Apoaequorin** | **B vit.** | **Tryptophan** | **Others** |
| --- | --- | --- | --- | --- | --- | --- | --- | --- | --- | --- | --- | --- |
| Araujo 2022 | Yes | 20 | 168 |  |  | 67.5 DHA  27 EPA |  |  |  |  |  | 75 pork brain  sphingolipids^a^ |
| Hadley 2017 | Yes | 19 | 175 |  |  | 26 DHA |  |  |  |  |  |  |
| Pan 2018 | Yes | 18 | 191 |  | 8.5 Vit. E  1.3 Vit. C | 33 DHA  37 EPA |  |  |  | 0.3 B1  0.2 B2  1.6 B3  0.5 B5  0.2 B6  0.06 B9  0.002 B12 |  | 390 Arginine |
| Milgram 2002 | Yes | 17 | 275 | 180 spinach flakes  180 tomato pomace  180 grape pomace  180 carrot granules  180 citrus pulp | 19 Vit. E  1.4 Vit. C |  | 4.7 L-carnitine  2.3 LA |  |  |  |  |  |
| Milgram 2015 | Yes | 17 | 32 |  |  |  |  |  | 0.25  0.5 |  |  |  |
| Pan 2010 | Yes | 17 | 225 |  |  |  |  |  |  |  |  | 1g^b^ MCT (5.5%) |
| Pero 2019 | Yes | 17 | 50 | 7.5 *Trametes versicolor*  2.5 *B. serrata Roxb. ex Colebr.*  2 *Harpagophytum procumbens*^c^  2 *Ginkgo biloba* leaves dry extract | 1.2 Vit. E | 15 Krill oil  5 Krill powder |  |  |  |  |  | 12.5 Glucosamine  1.5 CoQ10 |
| Araujo 2012 | Yes | 16 | 55 |  |  |  |  |  |  |  |  | 20 SAMe |
| Milgram 2004, 2005  Siwak 2005  Nippak 2007  (same clinical trial) | Yes | 16 | 1095 | 180 spinach flakes  180 tomato pomace  180 grape pomace  180 carrot granules  180 citrus pulp | 19 Vit. E  1.4 Vit. C |  | 4.7 L-carnitine  2.3 LA |  |  |  |  |  |
| Milgram 2015 | Yes | 16 | 32 |  |  |  |  |  | 0.5  1 |  |  |  |
| Snigdha 2016 | Yes | 15 | 1095 |  |  |  | 2.7 LA  5.4 ALCAR |  |  |  |  |  |
| Benedetti 2019 | Yes | 14 | 731 |  |  |  |  |  |  |  |  | 50 Homotaurine |
| Head 2012 | Yes | 14 | 275 | 40.6 green tea extract  3.4 *Piper nigrum* extract  40.6 curcumin |  |  | 10.2 LA |  |  |  |  | 17 NAC |
| Milgram 2007 | Yes | 14 | 76 |  |  |  | 11 LA  27.5 ALCAR |  |  |  |  |  |
| Araujo 2008 | Imp | 14.5 | 70*2 | 10 *Gingko biloba* extract | 6.7 Vit. E |  |  | 5 |  | 4.1 B6 |  |  |
| Chapagain 2020 | No | 24 | 365 | 8 green tea polyphenols | 16 Vit. E  11 Vit. C | 32 DHA |  | 6.3 |  |  | 86 |  |
| Fragua 2017 | No | 18 | 75 | group 1: 38.4 total polyphenols  group 2: 50.4 total polyphenols  (from PEGB) |  |  |  |  |  |  |  |  |
| Chapagain 2018^d^ | No | 17 | 365 | X | X | X |  | X |  |  | X |  |
| Christie 2009 | No | 16 | 129 |  |  |  | 11 LA |  |  |  |  |  |
| Christie 2009 | Worse  tend. | 16 | 129 |  |  |  | 27.5 ALCAR |  |  |  |  |  |
| Christie 2009 | No | 16 | 79 |  |  |  | 11 LA  27.5 ALCAR |  |  |  |  |  |
| Snigdha 2016 | No | 15 | 1095 | 180 spinach flakes  180 tomato pomace  180 grape pomace  180 carrot granules  180 citrus pulp | 18 Vit. E  1.4 Vit. C |  |  |  |  |  |  |  |
| Snigdha 2016 | No | 15 | 1095 | 180 spinach flakes  180 tomato pomace  180 grape pomace  180 carrot granules  180 citrus pulp | 18 Vit. E  1.4 Vit. C |  | 2.7 LA  5.4 ALCAR |  |  |  |  |  |
| Snigdha 2016 | Worse | 15 | 1095 |  |  |  | 2.7 LA |  |  |  |  |  |

Table S1: Dosages of nutrients administered in clinical trials evaluating cognitive function with a cognitive task in dogs.

Doses are calculated for a 10-kg dog and expressed as amount per kg of body weight per day. All amounts are in milligrams unless otherwise specified.

Imp: Improvement.

PEGB: polyphenol-rich extract from grape and blueberry.

QS: Quality Score, based on the quality score from the adapted CAMARADES checklist. Studies scoring 18 or above were classified as very high quality, those scoring 16 and 17 were considered good quality, those scoring 14 and 15 were categorized as medium quality, and studies scoring less than 14 were classified as low quality.

tend.: tendency.

Vit.: Vitamin(s).

X: Nutrient present but unspecified dosage.

^a^30% gangliosides, 35% sphingomyelins, 10% ceramids

^b^MCT: Medium Chain Triglycerides composed of 97% caprylic acid and 3% capric acid

^c^dry extract root

^d^”The exact composition of the diet cannot be revealed because it is protected by a confidential clause”

| **Trial ID** | **Effect** | **QS** | **Duration** | **Plant extracts and products** | **Vit. E / C** | **Omega 3 fatty acids** | **PS** | **Mitochondrial cofactors** | **CoQ10** | **B vit.** | **SAMe** | **Others** |
| --- | --- | --- | --- | --- | --- | --- | --- | --- | --- | --- | --- | --- |
| Dodd 2003^c^ | Yes | 18 | 60 | 0.03 β-carotene^a^ | 13 Vit. E  2.8 Vit. C^a^ | 170 omega-3 fatty acids^a^ |  | X |  |  |  |  |
| Reme 2008 | Yes | 15 | 60 |  |  |  |  |  |  |  | 18 |  |
| Heath 2007 | Yes | 14 | 42 |  | 2 Vit. E  4 Vit. C | 7 DHA EPA | 0.2 | 2 LA  1 ALCAR  2.7 L-Carnitine | 0.2 |  |  | 5 µg selenium  4 NAC |
| Pan 2018 | Imp | 23 | 90 |  | 6.5 Vit. E  2.7 Vit. C  5.6 Vit. E  2.4 Vit. C | 40 DHA  53 EPA  37 DHA  49 EPA |  |  |  | 1 B1  0.5 B2  4 B3  1.4 B5  0.3 B6  0.2 B9  0.003 B12  1 B1  0.5 B2  3.8 B3  1.4 B5  0.3 B6  0.2 B9  0.003 B12 |  | 315 Arginine  1.1g MCT (6.5%)  328 Arginine  1.5 g MCT (9%) |
| Lee 2022 | Imp | 13 | 90 | 2.1 Cyanidin-3-glucoside^b^ |  |  |  |  |  |  |  |  |
| Reichling 2006 | Imp | 10 | 56 | 4 *Ginkgo biloba* leaf extract |  |  |  |  |  |  |  |  |
| Dewey 2023^c^ | Imp | 8 | 90 | 27.2 curcumin  12.2 *Salvia miltiorrhizae*  6 *Polygala* *tenuifolia* | 6.4 Vit. E |  | 4.6 |  | 1 |  | 13.6 | Zinc 18.2 |
| Osella 2007 | Imp | 7 | 90 | 10 *Ginkgo biloba* | 6.7 Vit. E |  | 5 |  |  | 4.1 B6 |  |  |

Table S2: Dosages of nutrients administered in clinical trials evaluating cognitive function with a questionnaire in dogs.

Doses are calculated for a 10-kg dog and expressed as amount per kg of body weight per day. All amounts are in milligrams unless otherwise specified.

Imp: Improvement.

QS: Quality Score, based on the quality score from the adapted CAMARADES checklist. Studies scoring 18 or above were classified as very high quality, those scoring 16 and 17 were considered good quality, those scoring 14 and 15 were categorized as medium quality, and studies scoring less than 14 were classified as low quality.

Vit.: vitamin(s).

^a^Only dosage of vit. E provided, polyphenols, vitamin C and omegas-3 fatty acids are from the composition of Hill’s Prescription Diet B/D Brain Ageing.

^b^From honeyberry.

^c^No dosage in the article, composition of CogniCaps® found online on CityU Vet Boutique (https://www.cityuvb.com.hk/locale/en-US/product/VETDICATE-CogniCaps-60-Caps?srsltid=AfmBOoqqJBkDGS9zAG4mH6X29l7qV6AvEyxEn8hx3Ji6QBrqcHu8pvv3)

| **Trial ID** | **Effect** | **QS** | **Duration (d)** | **Vit. E / C** | **Omega 3 fatty acids** | **B vitamins** | **Others** |
| --- | --- | --- | --- | --- | --- | --- | --- |
| Pan 2013 | Yes | 18 | 345 | 8.4 Vit. E  1.2 Vit. C | 41 DHA  43 EPA | 0.8 B1  0.5 B2  0.8 B5  0.3 B6  0.06 B9  0.001 B12 | 345 Arginine |
| Araujo 2012 | Yes | 15 | 54 |  |  |  | 25 SAMe |

Table S3: Dosages of nutrients administered in clinical trials evaluating cognitive function with a cognitive task in cats.

Doses are calculated for a 4-kg cat and expressed as milligrams per kg of body weight per day.

QS: Quality Score, based on the quality score from the adapted CAMARADES checklist. Studies scoring 18 or above were classified as very high quality, those scoring 16 and 17 were considered good quality, those scoring 14 and 15 were categorized as medium quality, and studies scoring less than 14 were classified as low quality.

Vit.: Vitamin(s).
